# Artificial Intelligence in Acute Coronary Syndromes: From Early Diagnosis to Culprit Lesion Characterization, Coronary Physiology, and Risk Stratification

**DOI:** 10.3390/diagnostics16203429

**Published:** 2026-10-21

**Authors:** Marcello Marchetta, Lucio Giuseppe Granata, Branislav Crnomarkovic, Simona Giubilato, Giuseppe Andò, Mila Kovacevic

**Affiliations:** 1 Institute of Cardiovascular Disease of Vojvodina, 21204 Novi Sad, Serbia; 2 Department of Cardiology, Policlinico Tor Vergata, Viale Oxford, 81, 00133 Rome, Italy; 3 Cardiology Division, Garibaldi-Nesima Hospital, Azienda Ospedaliera di Rilievo Nazionale e di Alta Specializzazione (ARNAS) Garibaldi, 95122 Catania, Italy; 4 Cardiology Division, Cannizzaro Hospital, 95126 Catania, Italy; 5 Department of Cardiology, Azienda Ospedaliera Papardo, 98158 Messina, Italy

**Keywords:** artificial intelligence, machine learning, deep learning, acute coronary syndromes, culprit lesion, optical coherence tomography, coronary physiology, quantitative flow ratio

## Abstract

Artificial intelligence (AI) is being investigated across the acute coronary syndrome (ACS) care pathway, including early diagnosis, coronary lesion characterization, procedural guidance, and risk prediction. This comprehensive review examines the current evidence across the entire ACS trajectory, with particular emphasis on clinically actionable and interventional applications. AI-enabled electrocardiography (ECG) may improve detection of myocardial infarction, including occlusion myocardial infarction (OMI) that does not meet conventional STEMI criteria. Models combining clinical data and high-sensitivity troponin may improve early rule-out and rule-in. In coronary imaging, AI can quantify plaque characteristics and assist with the interpretation of OCT and IVUS, while image-derived physiology may help assess non-culprit lesions. However, better diagnostic accuracy does not necessarily translate into better revascularization decisions. AI-based models can also estimate post-ACS ischemic, bleeding, and mortality risk, although evidence that their use improves treatment or outcomes remains limited. The next priority is prospective evaluation of whether these tools improve decisions, workflow, and patient outcomes across diverse clinical settings.

## 1. Introduction

Ischemic heart disease remains a major global health burden [1]. Acute coronary syndrome (ACS) is its most unstable manifestation and retains substantial short- and long-term risk across healthcare systems [2]. Contemporary care requires rapid decisions based on symptoms, ECG, high-sensitivity cardiac troponin (hs-cTn), and, when indicated, coronary imaging and invasive assessment [3]. Yet each stage of this pathway leaves important uncertainties.

Conventional STEMI (ST-segment elevation myocardial infarction) criteria do not identify every acute coronary occlusion and may also prompt catheterization-laboratory activation in patients with non-ischemic ST-segment elevation patterns [4]. In an international evaluation, they identified only approximately one third of angiographically defined occlusion myocardial infarctions (OMI) [5]. After the culprit artery is identified, angiographic stenosis alone provides an incomplete picture of lesion-specific ischemia and the future risk of non-culprit plaques [6,7]. Intracoronary imaging adds valuable detail, but its interpretation is time-consuming and dependent on operator expertise [8,9].

Artificial intelligence (AI) offers ways to extract and combine information from ECGs, biomarkers, coronary images, and clinical data that may be difficult to capture through conventional assessment alone [10,11]. Technical accuracy, clinical utility, and patient benefit require separate evaluation [12,13]. Its proposed uses now extend from early diagnosis to plaque characterization, coronary physiology, procedural guidance, and risk prediction [5,14,15,16,17,18,19,20,21,22,23,24,25,26,27,28,29,30,31,32,33,34,35,36,37,38]. The central question is whether these capabilities improve the decisions clinicians make, rather than simply improving a model’s performance against a reference standard, particularly because much of the available evidence is retrospective or has yet to be tested in routine care [39].

**Figure 1:**
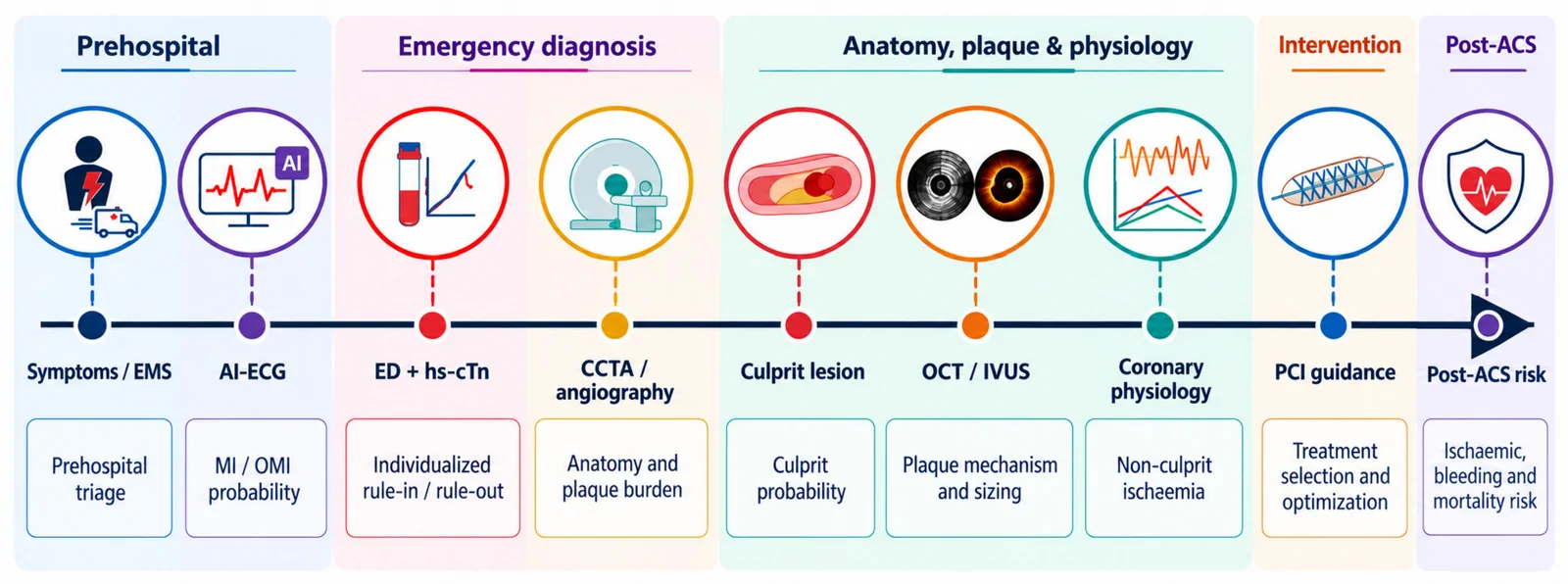
AI-enabled pathway in acute coronary syndromes. AI applications are mapped from symptoms and EMS-based prehospital triage, including AI-ECG, through emergency diagnosis, lesion characterization, PCI guidance, and post-ACS risk stratification. Abbreviations: ACS, acute coronary syndrome; AI, artificial intelligence; CCTA, coronary computed tomography angiography; ECG, electrocardiogram; ED, emergency department; EMS, emergency medical services; hs-cTn, high-sensitivity cardiac troponin; IVUS, intravascular ultrasound; MI, myocardial infarction; OCT, optical coherence tomography; OMI, occlusion myocardial infarction; PCI, percutaneous coronary intervention.

Recent broad reviews and American Heart Association statements have defined the general opportunities and requirements of cardiovascular AI, including validation, explainability, generalizability, and implementation [10,11,12,13]. Building on this foundation, the present review follows the successive decisions in ACS care, with particular attention to lesion assessment and interventional practice. Its distinguishing focus is the integration of culprit-versus-non-culprit lesion assessment, intracoronary imaging, image-derived physiology, and AI-assisted PCI within a single time-critical ACS pathway. For each application, we consider what AI adds to existing assessment, how well it has been validated, and whether its use has changed care or improved outcomes. Figure 1 summarizes the clinical pathway covered in this review.

## 2. Materials and Methods

This article was designed as a narrative review supported by a structured literature search; it was not intended as a systematic review or meta-analysis. PubMed/MEDLINE was searched from database inception through 25 July 2026. The search combined the ACS terms “acute coronary syndrome,” “myocardial infarction,” STEMI, NSTEMI, and “occlusion myocardial infarction” with the computational terms “artificial intelligence,” “machine learning,” “deep learning,” “neural network,” and “computer-aided.” These were combined with domain-specific terms covering electrocardiography, troponin, emergency triage, coronary computed tomography angiography, angiography, culprit lesion, plaque, optical coherence tomography, intravascular ultrasound, coronary physiology, fractional flow reserve, quantitative flow ratio, percutaneous coronary intervention, prognosis, and risk stratification. Searches were supplemented by backward reference screening of eligible articles, contemporary ACS guidelines, and relevant reviews and scientific statements [10,11,12,13], together with targeted forward citation searches for subsequent validation and implementation studies.

Titles and abstracts were screened for relevance, followed by full-text assessment of potentially eligible reports. We prioritized original human studies reporting clinically relevant diagnostic, procedural, workflow, or prognostic outcomes, particularly those including independent external or temporal validation, prospective evaluation, comparison with established clinical standards, or assessment of effects on clinical decision-making. Guidelines, scientific statements, systematic reviews, and methodological studies were included to provide clinical context and identify additional primary evidence. Studies outside dedicated ACS populations were retained selectively when they evaluated technologies directly transferable to ACS care, including automated intracoronary imaging, quantitative coronary angiography, and AI-enabled coronary physiology. Reports were excluded when they did not address an ACS-relevant clinical decision or a directly transferable coronary technology, lacked sufficient methodological or outcome information, or duplicated a more complete publication.

Evidence was synthesized qualitatively according to its position in the ACS pathway and stage of translation: model development and internal validation, independent external validation, prospective workflow implementation, modification of diagnosis or treatment, and demonstrated clinical benefit. Particular attention was paid to sample selection, reference standards, validation design, comparators, and clinical endpoints. Because the objective was a clinically integrated narrative appraisal of heterogeneous technologies rather than an exhaustive pooled estimate of a prespecified effect, no formal risk-of-bias instrument or meta-analysis was applied.

## 3. AI for Early Recognition of Acute Coronary Syndromes

### 3.1. Prehospital and Emergency Triage

The clinical value of early ACS recognition is intrinsically time-dependent, particularly in patients with an acutely occluded coronary artery, in whom delays in reperfusion increase irreversible myocardial injury [40,41]. The prehospital environment is therefore an attractive setting for AI because only a restricted set of variables is usually available, such as symptoms, vital signs, and electrocardiography, while decisions regarding transport destination, cath-lab activation, or escalation of care may have substantial consequences [40]. AI may help combine these limited inputs to identify patients who need faster assessment. The principal studies evaluating AI for early ACS recognition, ECG interpretation, biomarker integration, and acute triage are summarized in Table 1.

Al-Zaiti and colleagues provided one of the most clinically relevant early demonstrations of ML in this context. Rather than relying exclusively on conventional ST-segment criteria, their model extracted hundreds of temporal and spatial characteristics from the prehospital 12-lead ECG and tested its ability to predict ACS in independent prospective cohorts [14]. The principal contribution of this work was not merely an improvement in the area under the curve (AUC); it showed that quantitative information distributed across the ECG could recover sensitivity that is lost when interpretation is constrained to a limited number of predefined ischemic patterns. Importantly, the signal persisted beyond obvious STEMI, suggesting that AI-enhanced ECG interpretation may be most useful precisely in presentations in which the conventional tracing is diagnostically incomplete.

Takeda et al. pursued a complementary strategy by using information that can realistically be acquired by emergency medical services before arrival at the hospital. Their prospective multicenter model combined symptoms, vital signs, and three-lead ECG information, demonstrating that useful discrimination could be achieved even without a diagnostic 12-lead ECG or laboratory testing [42].

Goldschmied and colleagues extended this concept to the prediction of both ACS-related adverse events and acute coronary artery occlusion using variables available before hs-cTn assessment [43]. Although discrimination was more modest than in highly selected AI-ECG studies, the clinical target was arguably more pragmatic. Prehospital algorithms do not necessarily need to establish a final diagnosis; they may instead function as routing tools, identifying patients whose probability of an occlusive or otherwise high-risk coronary event warrants direct transfer to a PCI-capable center or accelerated invasive evaluation.

This evidence suggests that the principal prehospital application of AI may not be replacement of conventional diagnostic pathways, but probability enrichment before definitive testing becomes available. This has particular relevance in geographically remote systems, settings with prolonged transfer times, and regions in which immediate expert ECG interpretation is not consistently available. The same principle also limits the degree of confidence that should be assigned to such algorithms: because prevalence, ambulance referral patterns, and local thresholds for invasive treatment differ considerably among health systems, models derived in one emergency network may undergo significant dataset shift when deployed elsewhere [11,12]. External validation is therefore not an optional methodological refinement but a prerequisite for clinical implementation.

**Table 1:** Selected studies of AI for early ACS diagnosis, ECG interpretation, biomarker integration, and triage.

Study	Setting/Sample	Data & Model	Clinical Question	Main Finding	Clinical Relevance/Caveat
Than et al., 2019 [19]	Suspected MI; 11,011 patients	Age, sex, serial hs-cTnI; gradient boosting	Individualized type 1 MI probability	AUC 0.963; 69.5% low risk (NPV 99.7%); 10.6% high risk (PPV 71.8%).	Established the shift from fixed troponin thresholds to individualized probability; assay-specific and not initially tested in real-time care.
Al-Zaiti et al., 2020 [14]	Prospective prehospital cohorts; *n* = 1244	554 12-lead ECG features; ML fusion model	Prehospital ACS detection	Sensitivity increased by 52% vs. commercial ECG software and 37% vs. experienced clinicians, with high NPV.	Important proof that latent ECG information can improve prehospital triage; implementation across EMS systems remains untested.
Bouzid et al., 2021 [15]	Prospective EMS chest-pain cohort; *n* = 2400	557 ECG features; random forest	Angiographic culprit-lesion detection	AUC 0.85; sensitivity 71.1%, specificity 84.7%, NPV 96.1%.	Links ECG analysis to an actionable coronary endpoint rather than diagnostic coding alone.
W.-C. Liu et al., 2021 (AMI classifier) [18]	Retrospective single-center cohort; large ECG dataset	12-lead ECG ± cTnI; deep CNN	STEMI/NSTEMI detection and culprit-territory localization	STEMI AUC 0.997; for NSTEMI, ECG + cTnI AUC 0.978 vs. 0.877 for ECG alone.	Illustrates why multimodal integration is more important for NSTEMI; retrospective single-center design.
W.-C. Liu et al., 2021 (AI alarm strategy) [44]	Prospective validation: 14,296 ED visits; workflow comparison: 57 pre- vs. 32 post-implementation STEMI cases	Symptoms + ECG + hs-cTnI; AI alarm	AMI alerting and cath-lab activation	Validation: STEMI F-measure 0.932. Workflow: median ECG-to-activation 6 → 4 min and door-to-balloon 69 → 61 min.	Rare workflow evaluation; the single-center, non-randomized comparison with small STEMI cohorts precludes causal outcome inference.
Doudesis et al., 2022 [21]	Consecutive High-STEACS patients; *n* = 20,761	Age, sex, serial hs-cTnI; MI^3^	MI diagnosis and risk stratification	AUC 0.949; 62.5% low probability (NPV 99.8%) and 14.3% high probability (PPV 70.4%).	Large real-world validation supporting transportability within the same hs-cTnI platform.
Gustafsson et al., 2022 [45]	All-comer ED cohort; 214,250 patients/492,226 ECGs	ECG + age + sex; residual CNN ensemble	STEMI/NSTEMI detection	Random-test C-statistic 0.991 for STEMI and 0.832 for NSTEMI; temporal STEMI 0.985; external PTB-XL STEMI 0.932.	Very large real-world dataset; demonstrates the higher performance ceiling for STEMI than NSTEMI.
de Capretz et al., 2023 [46]	Consecutive ED chest pain; *n* = 9519	ECG + hs-cTnT + routine labs; LR/NN	Early 30-day AMI/death rule-in and rule-out	55.0% were ruled out and 5.3% ruled in at presentation while meeting prespecified safety and accuracy thresholds.	Shows practical value of combining electrical, biochemical, and routine clinical information; retrospective evaluation.
Doudesis et al., 2023 [20]	10,038 derivation; 10,286 external validation across 7 cohorts	Troponin + clinical variables; XGBoost	Individual probability of MI	AUC 0.953; 61% low probability at presentation vs. 27% with fixed troponin thresholds, with similar NPV.	One of the strongest externally validated diagnostic AI frameworks; randomized clinical-utility testing remains needed.
Herman et al., 2024 [5]	International EU/US testing; 3254 ECGs, 2263 contacts, 2222 patients; 489 OMI	Single 12-lead ECG; explainable AI model	Occlusion MI detection	AUC 0.938; sensitivity 80.6% vs. 32.5% for STEMI criteria; mean model-based detection time 2.3 vs. 5.3 h.	Clinically important challenge to STEMI-only triage; timing estimate was model-based, and prospective outcome benefit remains unproven.
Lopez-Ayala et al., 2024 [22]	Prospective multicenter APACE cohort; *n* = 6487	Serial hs-cTnI + age + sex; MI^3^	Early NSTEMI diagnosis vs. ESC 0/1 h algorithm	AUC 0.961; 64.5% low risk with sensitivity 99.1% and NPV 99.8%.	Robust external validation showing greater rule-out efficiency while preserving safety.
Toprak et al., 2024 [47]	Pooled prospective cohorts; *n* = 2560	Single POC hs-cTnI + 8 clinical variables	Immediate MI rule-out	At <0.5% probability, 35.1% ruled out; NPV 99.96%, sensitivity 99.68%.	Potentially moves personalized rule-out toward point-of-care settings; clinical and economic implementation not yet tested.
Cho et al., 2025 [17]	Derivation: 723,389 ECGs/300,627 patients; external: 261,429 ECGs/259,454 patients	Raw ECG; self-supervised transformer	AMI requiring revascularization	External AUROC 0.968 overall; STEMI 0.991; NSTEMI 0.947.	Large-scale self-supervised learning with a treatment-relevant endpoint; prospective workflow effect remains unknown.
Shroyer et al., 2025 [48]	95 physicians; enriched set of 18 ECGs (8 equivalents, 8 mimics, 1 STEMI, 1 normal; 50% OMI)	ECG interpretation; Queen of Hearts AI	Cath-lab activation for OMI-equivalent/mimic patterns	AI accuracy 88.9% vs. ~65.6% for physicians, with fewer missed OMI and inappropriate activations than emergency physicians.	Controlled reading study rather than clinical implementation; enriched prevalence and small ECG set limit real-world inference.

Abbreviations: ACS, acute coronary syndrome; AI, artificial intelligence; AMI, acute myocardial infarction; AUC/AUROC, area under the receiver operating characteristic curve; CNN, convolutional neural network; ECG, electrocardiogram; ED, emergency department; EMS, emergency medical services; hs-cTnI, high-sensitivity cardiac troponin I; hs-cTnT, high-sensitivity cardiac troponin T; MI, myocardial infarction; ML, machine learning; NPV, negative predictive value; NSTEMI, non-ST-segment elevation myocardial infarction; OMI, occlusion myocardial infarction; POC, point of care; PPV, positive predictive value; STEMI, ST-segment elevation myocardial infarction.

### 3.2. AI-Enabled ECG

DL has transformed electrocardiographic analysis because the ECG is a high-dimensional biological signal in which clinically relevant information is distributed across waveform morphology, temporal relationships, and combinations of leads [49]. Conventional interpretation necessarily compresses this information into a manageable set of recognized patterns. Neural networks, in contrast, can operate on the raw or minimally processed signal and identify representations that need not correspond directly to established human descriptors [50].

Early proof-of-concept studies, including that of Chen et al., established the feasibility of detecting MI in selected ECG datasets [16]. Results from such datasets, however, may not reflect performance among consecutive emergency-department patients with pre-existing ECG abnormalities and competing diagnoses.

More clinically representative evidence emerged from much larger real-world datasets. Gustafsson et al. trained and tested a DL model using hundreds of thousands of ECGs from consecutive emergency-department patients and demonstrated near-excellent discrimination for STEMI, while NSTEMI detection remained more challenging [45]. This difference is clinically plausible: NSTEMI may have subtle or transient ECG changes, and its diagnosis also depends on evidence of myocardial injury.

The study by Wen-Cheng Liu et al. provides a particularly clear illustration of this distinction [18]. Their DL model achieved extremely high discrimination for STEMI and could also infer the infarct-related coronary territory, but its standalone performance for NSTEMI was substantially lower. Importantly, combining the ECG model with cardiac troponin markedly improved NSTEMI identification.

Cho et al. subsequently demonstrated what can be achieved when model scale and representation learning are expanded substantially. They used a different, clinically relevant endpoint—MI requiring revascularization—and externally validated their ECG model [17]. The use of an angiographically relevant therapeutic endpoint is particularly important. Models trained merely to reproduce diagnostic codes may inherit ambiguities in the labels themselves; models linked to revascularization-requiring infarction move the prediction target closer to the clinical decision that matters in acute care.

Multimodal DL represents another logical extension [49,51]. A study integrated ECG data with age, sex, chest pain, and dyspnea, showing that clinical context provided incremental information beyond the ECG signal alone [49]. Together, these studies suggest that the prediction target and the available clinical information matter as much as model complexity. High performance in a selected ECG dataset should not be assumed to persist among consecutive patients with competing diagnoses or pre-existing ECG abnormalities.

### 3.3. AI and the Identification of Occlusion Myocardial Infarction

Perhaps the most clinically consequential evolution of AI-ECG is the transition from automated STEMI recognition toward identification of occlusion myocardial infarction (OMI). The distinction is not semantic. STEMI describes an ECG pattern while OMI refers to an acute coronary lesion whose recognition may call for urgent reperfusion. When the two overlap, the traditional paradigm functions well. When they diverge, a patient with acute coronary occlusion may be managed as NSTEMI and experience substantial delays before angiography [52].

In an international evaluation, an AI-ECG model detected approximately 81% of angiographically defined OMI, compared with approximately 33% identified by STEMI criteria, while maintaining specificity above 90% [5]. More importantly, among patients with OMI missed by STEMI criteria, the AI model correctly identified a substantial proportion using the first ECG. This suggests a potential opportunity for earlier recognition, although the study did not test whether AI-guided triage shortens time to treatment or improves outcomes.

In a separate reading study, AI classified a selected set of difficult STEMI-equivalent and STEMI-mimic ECGs more accurately than emergency physicians and cardiologists, reducing both missed occlusions and inappropriate cath-lab activation [48]. However, the design does not establish real-world clinical superiority because OMI prevalence was artificially high and physicians were deprived of the broader clinical context ordinarily available in practice.

Rather than reporting only whether STEMI criteria are met, a future ECG report might also provide an estimated probability of acute coronary occlusion [5,50,53]. How clinicians should act on that estimate must be tested prospectively, taking into account symptoms and serial ECGs, as well as missed occlusions, unnecessary cath-lab activations, time to reperfusion, and patient outcomes. An illustrative report also described AI-assisted recognition of proximal left anterior descending artery occlusion in a patient with bifascicular block [54], but a single case cannot establish diagnostic performance.

### 3.4. From Diagnostic Performance to Workflow Impact

For diagnostic AI, the key translational question is whether a prediction is delivered early enough and in a form that changes care. Liu et al. evaluated an AI-based alarm that combined symptoms, ECG, and high-sensitivity troponin within an emergency workflow [44]. Following implementation, median ECG-to-cath-lab activation time fell from 6 to 4 min and door-to-balloon time from 69 to 61 min. This provides an example of the potential workflow benefit. Because the comparison was conducted at one center before and after implementation, it cannot establish that the alarm caused these improvements or improved patient outcomes.

## 4. AI-Enabled Biomarkers and Personalized Diagnostic Integration

### 4.1. From Fixed Troponin Thresholds to Individualized Probability

Hs-cTn enables rapid assessment of suspected myocardial infarction (MI) and underpins accelerated rule-out and rule-in pathways [55,56,57]. Its interpretation nevertheless depends on clinical context, including age, sex, renal function, time from symptom onset, and serial change [58,59,60]. AI models can combine these factors to estimate an individual patient’s probability of MI rather than relying on a troponin concentration alone [61,62,63].

The myocardial-ischaemic-injury index (MI^3^) introduced by Than et al. uses age, sex, and serial hs-cTnI measurements to estimate the probability of type 1 MI [19]. Its performance has been evaluated in large cohorts, including more than 20,000 consecutive patients [21]. In a multicenter comparison with the ESC 0/1 h algorithm, MI^3^ assigned more patients to rule-out, whereas the guideline algorithm achieved slightly higher sensitivity and negative predictive value [22]. This illustrates the clinically relevant trade-off between diagnostic efficiency and safety at a chosen threshold.

The Collaboration for the Diagnosis and Evaluation of Acute Coronary Syndrome (CoDE-ACS) model represents a broader version of individualized diagnosis. Rather than focusing predominantly on hs-cTn kinetics, it integrates hs-cTn concentrations with routinely available clinical information, including age, sex, sampling time, ECG findings, renal function, hematologic variables, and cardiovascular history [20]. Developed in more than 10,000 patients and externally validated in a similarly large population across seven international cohorts, it produces an individualized probability of MI rather than assigning patients solely according to fixed troponin thresholds. At presentation, a prespecified low-probability score identified 61% of patients as having a low probability of MI, compared with 27% using fixed troponin thresholds, with a similar negative predictive value. These findings are promising, but thresholds and calibration must be assessed in the population and clinical workflow where the model will be used.

### 4.2. Combining Tests and Moving Diagnosis Closer to the Patient

ECG and troponin provide complementary information: the ECG reflects electrical changes associated with ischemia, whereas troponin indicates myocardial injury. Their combination may therefore aid the assessment of suspected NSTEMI when the presenting ECG is inconclusive [18,64]. Models incorporating ECG information, hs-cTnT, age, sex, and routine laboratory variables have been developed to estimate the 30-day composite risk of AMI or all-cause death. In de Capretz et al., the best neural-network model ruled out 55.0% and ruled in 5.3% of patients at presentation while meeting prespecified safety and accuracy thresholds [46]. Similarly, combining a DL ECG model with troponin was shown to substantially improve NSTEMI discrimination compared with either modality alone [18]. Class imbalance in unselected chest-pain cohorts can make overall accuracy misleading; sensitivity, precision, F1 score, and AUROC should therefore be reported alongside accuracy [65].

Point-of-care testing may bring individualized assessment closer to the time of presentation. Using a single point-of-care hs-cTnI measurement, the ARTEMIS algorithm classified approximately one third of patients as suitable for immediate rule-out at a prespecified probability threshold [47]. The proportion ruled out was more than twice that achieved by conventional ESC or ACC single-sample pathways [3,66]. Although the patients came from prospective cohorts, this was a retrospective analysis in which the model did not direct management. Prospective studies must establish whether its use maintains safety and improves patient flow, particularly outside the emergency department.

## 5. AI for Coronary Plaque Characterization and Culprit Lesion Identification

### 5.1. Beyond Luminal Stenosis: Quantitative Plaque Phenotyping

Coronary artery disease has traditionally been assessed primarily by luminal stenosis severity. In ACS, however, stenosis severity does not fully capture plaque instability: a future culprit lesion may be only moderately obstructive before the event, while a severe stenosis may remain clinically silent [8,67]. Coronary computed tomography angiography (CCTA) can assess plaque burden, composition, remodeling, and calcification alongside luminal narrowing [68]. AI enables these features to be quantified across lesions and vessels at a scale that would be difficult to achieve by manual analysis [13,69].

A prerequisite for clinically meaningful AI-based plaque characterization is accurate and reproducible quantification. In a prospective comparison involving 237 clinically stable patients and 432 lesions, automated CCTA analysis showed strong agreement with intravascular ultrasound for total, calcified, and non-calcified plaque volumes [69]. This validates plaque quantification, but accurate segmentation is not itself evidence that an algorithm can predict ACS. Clinical value requires further demonstration that these measurements improve lesion-level prediction and, ultimately, patient management. Key studies applying AI and related computational methods to coronary plaque characterization, intracoronary imaging, coronary physiology, and procedural assessment are summarized in Table 2.

### 5.2. Identifying Culprit Lesions: Morphology, Physiology, and Inflammation

The identification of the culprit lesion in ACS is usually straightforward when a single severe stenosis with angiographic thrombus or occlusion is present. Diagnostic uncertainty increases substantially in multivessel disease, particularly in NSTEMI where multiple intermediate or severe lesions may coexist and angiographic signs of instability may be absent [8,67]. AI-based CCTA models address this problem by combining anatomical severity with plaque composition and lesion-specific hemodynamics [26,71]. In the EMERALD-II analysis, patients who had undergone CCTA between one month and three years before a subsequent ACS event were evaluated at the lesion level, with culprit status adjudicated from invasive angiography. Addition of AI-derived quantitative plaque and hemodynamic parameters to a reference model based on stenosis severity and conventional high-risk plaque features improved discrimination for future culprit lesions. Variables related to pressure loss across the lesion, plaque burden, total plaque volume, low-attenuation plaque, and downstream myocardial blood-flow characteristics provided information beyond the conventional anatomical model [26].

The clinical implication is that plaque vulnerability is unlikely to be encoded in a single morphological feature. Low-attenuation plaque, positive remodeling, and plaque burden provide information about plaque biology and architecture, whereas lesion-specific pressure gradients and flow characteristics describe the interaction between plaque geometry and coronary hemodynamics [73]. A multicenter model incorporating plaque and CT-derived physiological information also identified culprit lesions in patients with established ACS, with an AUC of approximately 0.85 in independent testing; when applied to prospective cohorts, the derived phenotype provided more modest stratification of future ACS risk [71]. These are related but distinct tasks: identifying the culprit during an event and estimating which plaque may cause a later event.

Local inflammation, vascular remodeling, mechanical forces, and lesion-specific hemodynamics interact with plaque composition and systemic risk factors to determine whether a plaque remains quiescent, progresses, or becomes clinically unstable [74]. Consequently, models based exclusively on luminal stenosis or plaque morphology are biologically incomplete. CCTA offers the unusual possibility of interrogating several of these domains non-invasively within the same examination, including plaque composition, CT-derived physiology, and pericoronary adipose tissue characteristics [13,25].

A lesion-level analysis integrating plaque characteristics, CT-derived physiological indices, and pericoronary adipose tissue attenuation demonstrated that models combining these domains discriminated culprit lesions more effectively than more restricted approaches. Variables related to minimal luminal area and lesion length captured anatomical severity; low-attenuation and fibrous plaque quantified composition; CT-FFR and translesional pressure change represented functional significance; and pericoronary fat attenuation provided a surrogate of the local inflammatory environment. The best-performing integrated model achieved an AUC of approximately 0.82, supporting the principle that information from anatomically adjacent but biologically distinct compartments may be complementary [25]. The same study also provides an important methodological caution. The random-forest candidate showed near-perfect discrimination in training but considerably weaker performance in the test dataset, whereas simpler models generalized more consistently [12,25].

These findings also suggest that the optimal role of AI is likely to differ according to disease stage. In established ACS, integration of plaque morphology, hemodynamics, and inflammatory features may assist with culprit identification when angiography is ambiguous [8]. Before ACS develops, similar information may identify plaques requiring more intensive systemic prevention rather than focal intervention. The distinction is clinically crucial. Although PREVENT demonstrated benefit from preventive PCI in carefully selected imaging-defined, non-flow-limiting vulnerable plaques, those findings should not be generalized to every plaque labelled high risk by an AI model [75]. The immediate therapeutic consequence of AI-based risk prediction may therefore be intensified lipid lowering, risk-factor modification, or closer surveillance rather than pre-emptive stenting of every high-risk lesion [26,71,75].

### 5.3. From Plaque Evolution to Residual Coronary Risk

Most coronary risk models are derived from a single imaging time point, although atherosclerosis evolves over time. In a matched serial-CCTA analysis, lesions that subsequently became culprits showed adverse changes in plaque characteristics and CT-derived physiology; a model incorporating these changes achieved an AUC of 0.918 [24]. In a separate CCTA study of 140 patients and 165 unrevascularized non-culprit intermediate lesions, low-attenuation plaque independently predicted lesion-volume progression and major adverse cardiovascular events [76]. Together, these findings suggest that the plaque characteristics and their evolution may refine risk assessment. They do not, however, justify routine repeat CCTA solely for AI-based surveillance, given the additional imaging burden and the absence of evidence that surveillance-guided treatment improves outcomes [10,24,76].

After ACS, risk also extends beyond the treated culprit lesion. Multiple high-risk plaques may coexist, and the lesion responsible for a future event may not be the one with the greatest baseline stenosis [77]. AI-based assessment of residual plaque burden and lesion-specific features across the coronary tree could therefore connect plaque-level findings with patient-level risk [26,71,77]. At present, however, AI-CCTA is best supported as a tool for quantitative phenotyping and risk assessment. Prospective studies must determine whether its findings can guide prevention or intervention in ways that improve clinical outcomes.

## 6. AI and Intracoronary Imaging in Acute Coronary Syndromes

Intracoronary imaging provides spatial resolution and tissue information that cannot be obtained from angiography alone. Optical coherence tomography (OCT) can directly visualize fibrous-cap morphology, thrombus, lipid-rich plaque, calcification, plaque rupture, and OCT-defined erosion [78], while IVUS provides robust assessment of vessel dimensions, plaque burden, remodeling, and calcium distribution [79]. The principal limitation in an acute setting is not lack of information but the opposite: a single pullback contains hundreds of frames that must be interpreted rapidly during an invasive procedure. AI is therefore uniquely attractive because the task involves high-dimensional image sequences in a time-sensitive environment where reproducibility and speed are clinically relevant [27,29].

Traditional automated image-analysis tools have largely focused on segmentation, identifying the lumen, vessel boundaries, or calcium. DL allows a transition from geometrical segmentation toward pathobiological classification, potentially identifying plaque phenotypes associated with mechanisms of ACS. This distinction matters because an algorithm that automatically measures lumen area improves efficiency, whereas an algorithm that differentiates plaque rupture from erosion or identifies thin-cap fibroatheroma (TCFA) may influence mechanistic interpretation and potentially treatment strategy [29,30].

### 6.1. Automated IVUS Analysis

In a 598-vessel study, automated classification achieved approximately 93% frame-level accuracy for attenuated plaque and 96% for calcification. Quantified attenuation and calcium burdens correlated strongly with human measurements, while inference required only approximately 0.05 s per frame and fewer than eight seconds for an entire vessel. These times are clinically relevant: if integrated into the imaging console, automated analysis could theoretically return quantitative plaque and calcium information while the operator is still planning the intervention [27].

A separate DL framework using cascaded U-Net architectures demonstrated automated segmentation of lumen, media-adventitia boundaries, and calcification, providing a technical foundation for downstream quantitative IVUS assessment [28]. The clinical importance of such segmentation lies less in the segmentation itself than in what becomes possible once reliable vessel geometry is available: automatic plaque-burden calculation, calcium arc and length assessment, reference-vessel sizing, lesion-length estimation, and potentially recommendation of lesion-modification strategies. Nevertheless, these possibilities remain predominantly inferential because available studies have evaluated image-analysis performance rather than AI-directed PCI outcomes.

The potential procedural relevance is particularly evident for calcified disease. The severity, circumferential distribution, and longitudinal extent of calcium influence stent expansion and may determine the need for rotational atherectomy, intravascular lithotripsy, or other plaque-modification techniques [80]. An AI system capable of automatically identifying calcium phenotype and integrating it with vessel dimensions could support procedural planning and reduce operator-dependent variability. However, the current evidence does not establish that algorithm-derived calcium quantification improves stent expansion or clinical outcomes, and this distinction should prevent extrapolation beyond the validated task [27].

### 6.2. Deep-Learning Identification of Thin-Cap Fibroatheroma

TCFA represents a structurally vulnerable plaque phenotype characterized by a lipid-rich necrotic core covered by a thin fibrous cap [81]. OCT is uniquely suited to its in vivo identification because its axial resolution permits measurement of cap thickness, but manual evaluation over an entire pullback is time-consuming and subject to reader variability. Automated detection therefore provides an archetypal use case for image-based AI: the pathological feature is visually definable, clinically relevant, and repeated across many sequential frames [30].

A DenseNet-based model trained on 45,400 OCT frames from 602 coronary lesions achieved an AUC of approximately 0.96 for TCFA detection and close agreement with expert estimates of vessel-level TCFA burden. Importantly, Grad-CAM localization suggested that the model focused on the thin-cap region in the vast majority of correctly classified TCFA frames, providing a degree of biological interpretability rather than purely opaque classification. Whole-pullback analysis required only a few seconds. Automated frame-level analysis makes quantitative assessment of TCFA feasible and could allow a continuous measure of vulnerable-plaque burden. Whether such quantification adds prognostic information or modifies therapy after ACS remains to be established, but conceptually it demonstrates how AI can convert a labor-intensive categorical imaging diagnosis into a scalable continuous phenotype [30].

### 6.3. Plaque Erosion

Plaque erosion is particularly important because it may have direct therapeutic implications. Unlike plaque rupture, erosion is characterized by an intact fibrous cap with overlying thrombus [82], and selected patients with OCT-defined erosion have been investigated using antithrombotic therapy without routine stent implantation [83]. However, the diagnosis of plaque erosion requires careful review of consecutive frames to ensure that rupture is not present, making interpretation difficult for less experienced operators during emergency PCI. This sequential nature of the task exposes a limitation of conventional convolutional neural networks, which generally interpret a selected frame without explicitly modelling its relationship with neighboring frames. A Transformer architecture was therefore developed to integrate information across consecutive OCT images, conceptually emulating the manner in which an experienced reader scrolls backward and forward through a pullback. In external validation, the sequential Transformer substantially outperformed the conventional CNN for both frame-level and lesion-level plaque-erosion diagnosis, with lesion-level AUC approximately 0.91 [29]. The reader-assistance experiment may be even more clinically relevant than the standalone AUC. When the AI output was made available to a less experienced OCT reader, diagnostic sensitivity increased substantially and approached that of a highly trained reader. This suggests a role for AI as an expertise equalizer: rather than replacing intracoronary imaging expertise, an algorithm could reduce the performance gap between high-volume imaging centers and cath labs in which complex OCT phenotyping is performed less frequently.

The therapeutic implications nonetheless require restraint. Even perfect automated identification of OCT-defined erosion would not by itself prove that a no-stent strategy is appropriate for all patients classified by the algorithm. Treatment decisions also depend on residual stenosis, thrombus burden, flow, clinical stability, antithrombotic risk, and the evidence supporting conservative management [84]. Thus, AI may first improve the reliability of mechanistic diagnosis; whether this should trigger a different intervention is a separate clinical question requiring dedicated prospective trials.

### 6.4. Layered Plaque and the Detection of Healed Coronary Instability

Layered plaque provides a different biological perspective on ACS. Rather than identifying the mechanism of the current acute event, layered structures are thought to reflect previous episodes of plaque disruption and healing, thereby encoding the history of coronary instability within vessel morphology [85]. Recognition can be subtle because signal patterns may extend across adjacent frames and can be difficult to distinguish from normal vessel-wall architecture.

A Vision Transformer developed for this task used attention mechanisms and inter-frame relationships to identify layered plaque more accurately than a conventional single-frame CNN. The conceptual importance parallels that of plaque-erosion detection: when the diagnostic target is inherently volumetric or sequential, algorithms that preserve relationships across the pullback are likely to be more appropriate than frame-isolated classifiers. External validation supported generalizability, although expert OCT interpretation rather than histology necessarily served as the reference standard [31].

Identification of healed or layered plaque may eventually contribute to a broader measure of cumulative plaque instability. A coronary segment containing multiple healed disruptions, extensive lipid-rich plaque, and recurrent thrombosis may carry different biological implications from a morphologically similar stenosis without evidence of prior instability. At present, however, this remains primarily a phenotyping application. There is insufficient evidence to use AI-detected layered plaque as an independent indication for PCI or modification of antithrombotic therapy.

### 6.5. AI-Supported Mechanistic Phenotyping of ACS

Taken together, automated detection of TCFA, erosion, attenuation, calcification, and layered plaque suggests a future in which intracoronary imaging is no longer interpreted only through a sequence of manual measurements. A single OCT or IVUS pullback could theoretically generate a structured report containing lumen and vessel dimensions, plaque burden, calcium phenotype, lipid or attenuation burden, cap characteristics, rupture/erosion probability, healed plaque burden, and procedural optimization metrics. The output would therefore describe not just where the stenosis is, but what biological process the lesion represents [27,29,30,31].

The greatest clinical value may arise from combining mechanistic classification with procedural decision support. For example, plaque erosion may support consideration of a conservative strategy in carefully selected cases; extensive calcium may prompt plaque modification; large lipid or attenuated-plaque burden may identify risk of distal embolization; and automated vessel sizing may refine stent selection. These are distinct tasks and should not be collapsed into a single generic “AI-guided PCI” concept. Each requires its own validation against an appropriate reference and, ultimately, demonstration that acting on the algorithmic output improves procedural or clinical outcomes.

## 7. AI-Enabled and Computational Coronary Physiology in Acute Coronary Syndromes

### 7.1. The Physiological Problem of the Non-Culprit Lesion

Multivessel coronary disease is common in ACS, making management of non-culprit stenoses a central interventional problem [86,87]. Once the culprit vessel has been treated, angiography frequently reveals one or more additional lesions of intermediate severity. Visual estimation alone cannot reliably determine whether these stenoses cause myocardial ischemia, while indiscriminate treatment risks unnecessary PCI and leaving functionally important lesions untreated may contribute to recurrent events. Contemporary ACS management therefore requires a distinction between anatomical completeness and physiological appropriateness of revascularization. Randomized evidence, most notably the COMPLETE trial, supports staged complete revascularization in appropriately selected patients with STEMI and multivessel disease [3,88,89,90].

Pressure-wire FFR remains an established reference for lesion-specific physiological assessment, but its application in ACS presents practical and biological complexities [91,92]. Hyperemic indices may be influenced by transient microvascular dysfunction, particularly in the infarct-related territory, while additional wire manipulation and hyperemic agents prolong the invasive procedure [93]. In non-culprit vessels, the physiological environment is generally more stable, yet operators may still defer assessment because of procedure duration, patient condition, contrast exposure, or a preference for staged evaluation [94]. These constraints provide the rationale for computational physiology derived from images already acquired during CCTA or angiography.

An important terminological distinction is necessary. Not all image-derived physiology is artificial intelligence. Some QFR approaches rely principally on three-dimensional quantitative angiography and mathematical flow modelling; other CT-FFR and angioFFR systems explicitly use machine-learning models trained on synthetic computational-fluid-dynamics datasets. For this reason, the clinically relevant category is better described as AI-enabled and computational coronary physiology, while the underlying methodology should be specified for each system rather than treating all wire-free indices as equivalent AI applications [23,32,34].

### 7.2. Machine-Learning CT-FFR

ML CT-FFR provides a particularly direct link between AI and coronary physiology. The underlying approach can be trained on large databases of synthetic coronary anatomies for which pressure and flow have been calculated through computational fluid dynamics. Once trained, the model estimates lesion-specific pressure loss from geometric features of a patient’s CCTA without repeating computationally intensive full CFD simulations for each case [23].

An early ACS study used this approach to interrogate 81 non-culprit lesions in 48 patients with multivessel disease. Despite the lesions having been considered non-critical at index invasive angiography, approximately 28% were classified as physiologically significant by ML-based CT-FFR. During follow-up, CT-FFR ≤ 0.80 was associated with subsequent major adverse cardiac events and provided incremental prognostic information beyond clinical risk and anatomical CAD-RADS classification [23]. Functional significance may identify a non-culprit lesion that is already capable of producing ischemia, but future ACS can also arise from biologically vulnerable plaques that are not severely flow-limiting at baseline. Consequently, physiology and vulnerability should not be viewed as competing methods. They describe different dimensions of lesion risk: ischemic burden and propensity to acute destabilization. The most promising AI frameworks are therefore those capable of integrating both rather than reducing residual coronary risk to a single FFR threshold [23,26].

### 7.3. Angiography-Derived Physiology

Angiography-derived physiological indices offer a different practical advantage: they can be calculated from images acquired during the index invasive procedure, potentially without a pressure wire or additional hyperemic medication [95]. This is particularly attractive in ACS because an intermediate non-culprit lesion may only become a management question after the acute culprit intervention has been completed. Computational analysis can theoretically be performed offline, allowing the operator to reconsider the functional relevance of residual disease without repeating invasive instrumentation [96]. In patients with acute MI and multivessel disease, QFR derived from index angiography demonstrated good agreement with subsequent functional testing performed using invasive FFR, stress CMR, or SPECT. In 220 patients with 280 non-culprit vessels, discrimination for functional significance was high, with an AUC close to 0.89, and QFR outperformed angiographic measures of stenosis severity. A proposed low- and high-probability strategy suggested that a substantial proportion of lesions could theoretically have been classified without staged ischemia testing [6].

These findings demonstrate diagnostic feasibility but also expose practical dependencies. Retrospective QFR analysis requires angiographic projections of adequate quality, limited vessel overlap, sufficient contrast filling, and appropriate acquisition geometry [95,97]. In the acute setting, angiography is performed primarily to identify and treat the culprit lesion rather than to optimize every projection for subsequent computational physiology. A meaningful proportion of lesions may therefore be non-analyzable because the images required by the algorithm were never acquired. This difference between algorithm performance under analyzable conditions and population-level feasibility is critical when assessing real-world implementation [6,34].

### 7.4. AI-Based angioFFR

A more explicitly AI-based angiographic approach has used supervised ML trained on more than 27,000 synthetic coronary geometries with CFD-derived pressure values. In clinical use, two angiographic views are reconstructed into a patient-specific coronary model, and the trained algorithm estimates pressure loss along the vessel. This represents a different computational paradigm from solving full fluid-dynamic equations de novo for every patient: the physiological relationship is learned upstream and then rapidly applied at the point of care.

Prospective validation against invasive FFR demonstrated an overall accuracy of 87.7%, sensitivity of 76.8%, specificity of 94.3%, and AUC of 0.90, outperforming conventional 2D-QCA for identifying FFR-defined hemodynamically significant stenoses. The pullback-derived pressure gradients were also comparable with invasive FFR gradients in pre-stenting segments, suggesting that AI-based angiographic physiology may potentially provide not only a distal binary value but spatial information about where pressure loss occurs [32].

The major limitation for the present review is that this validation population was predominantly non-AMI (57.0% stable angina, 32.5% silent ischemia, and only 10.5% unstable angina), and MI within 72 h was excluded. The study therefore validates the technology rather than its ACS-specific application. This distinction should be maintained because model performance in stable coronary physiology cannot automatically be extrapolated to the dynamic microvascular and hemodynamic conditions of acute MI.

### 7.5. CT-FFR Before Invasive Angiography in High-Risk NSTE-ACS

The use of CT-derived physiology before invasive angiography offers a different workflow concept. Rather than analyzing non-culprit lesions after angiography, CCTA with CT-FFR can potentially provide a physiological map before the patient enters the cath lab [98]. In high-risk NSTE-ACS, this could theoretically identify which additional vessels are unlikely to contain functionally significant disease and reduce the need for pressure-wire interrogation during the invasive procedure.

In a prespecified post hoc analysis of a multicenter study, 49 patients with high-risk NSTE-ACS and 67 angiographically identified non-culprit lesions were evaluated using CT-FFR against invasive FFR. CT-FFR achieved 93% sensitivity, 79% specificity, and an overall diagnostic accuracy of 85%, with a negative predictive value (NPV) of 94%. The high NPV is clinically important because the technology may be better suited to ruling out significant residual disease than to definitively identifying every lesion that requires PCI; the more modest positive predictive value suggests a risk of overestimating lesion significance [33].

The strategy nevertheless faces substantial logistical barriers. High-risk NSTE-ACS patients are frequently referred directly for invasive angiography, and adding CCTA requires additional contrast, imaging capacity, and coordination without proven improvement in clinical outcomes. Therefore, the value proposition is likely to be patient-selective rather than universal. CT-FFR may be most attractive when CCTA is already clinically indicated or has already been performed, allowing physiological information to be extracted from an existing dataset rather than introducing an additional mandatory test before angiography.

### 7.6. From Diagnostic Accuracy to Revascularization Decisions: The Lesson of Randomized Evidence

The ultimate clinical test of any physiological technology is not whether it correlates with invasive FFR, but whether using it changes treatment appropriately and improves outcomes. This distinction is particularly important in ACS, where the decision to treat a non-culprit lesion depends on multiple factors beyond its numerical physiological value, including lesion morphology, vessel size, territory at risk, timing of revascularization, clinical stability, and the broader evidence supporting complete revascularization [3].

A randomized study directly compared QFR-guided and angiography-guided management of non-culprit lesions after successful culprit PCI in 202 patients with STEMI, NSTEMI, or unstable angina and multivessel disease. Contrary to the prespecified hypothesis, QFR guidance did not significantly alter the proportion of patients assigned to PCI versus medical treatment and did not reduce the composite clinical endpoint at 12 months [34].

This neutral result is scientifically important. The failure of a physiologically sophisticated strategy to improve clinical decisions does not invalidate QFR as a measurement tool. Instead, it demonstrates that measurement accuracy and decision utility are different constructs. If angiographic interpretation by experienced operators already results in a relatively conservative and appropriate strategy, adding QFR may produce little reclassification. Furthermore, physiological significance is only one component of residual lesion risk, and a stenosis with QFR above 0.80 may still harbor a vulnerable plaque capable of producing a future ACS event [26,34].

This observation has direct implications for AI development. Future systems should not be designed merely to reproduce FFR with increasing numerical precision. The more ambitious clinical target is to determine which non-culprit lesion should be treated, deferred, or medically intensified, integrating physiology with plaque morphology, myocardial territory, procedural risk, and patient-level context. Such a system would move beyond “virtual FFR” toward actual clinical decision support, but it would also require a substantially higher evidentiary standard because its output would directly influence revascularization [26,71].

### 7.7. Integrating Functional Significance and Plaque Vulnerability

The traditional dichotomy between anatomical and physiological lesion assessment is inadequate for ACS. A stenosis may be functionally significant without being biologically unstable, while a non-flow-limiting plaque may carry substantial future thrombotic risk [7,99]. A comprehensive non-culprit assessment should therefore distinguish ischemia-producing lesions, vulnerable lesions, and lesions displaying both features. AI can integrate heterogeneous information including stenosis geometry, plaque volume and composition, CT-FFR or angiography-derived physiology, local pressure gradients, perivascular inflammation, and clinical variables. Current studies indicate that combined anatomical and hemodynamic models outperform stenosis-only approaches for culprit-lesion prediction [25,26,71], while serial imaging suggests that temporal deterioration adds information beyond a single baseline measurement [24].

**Figure 2:**
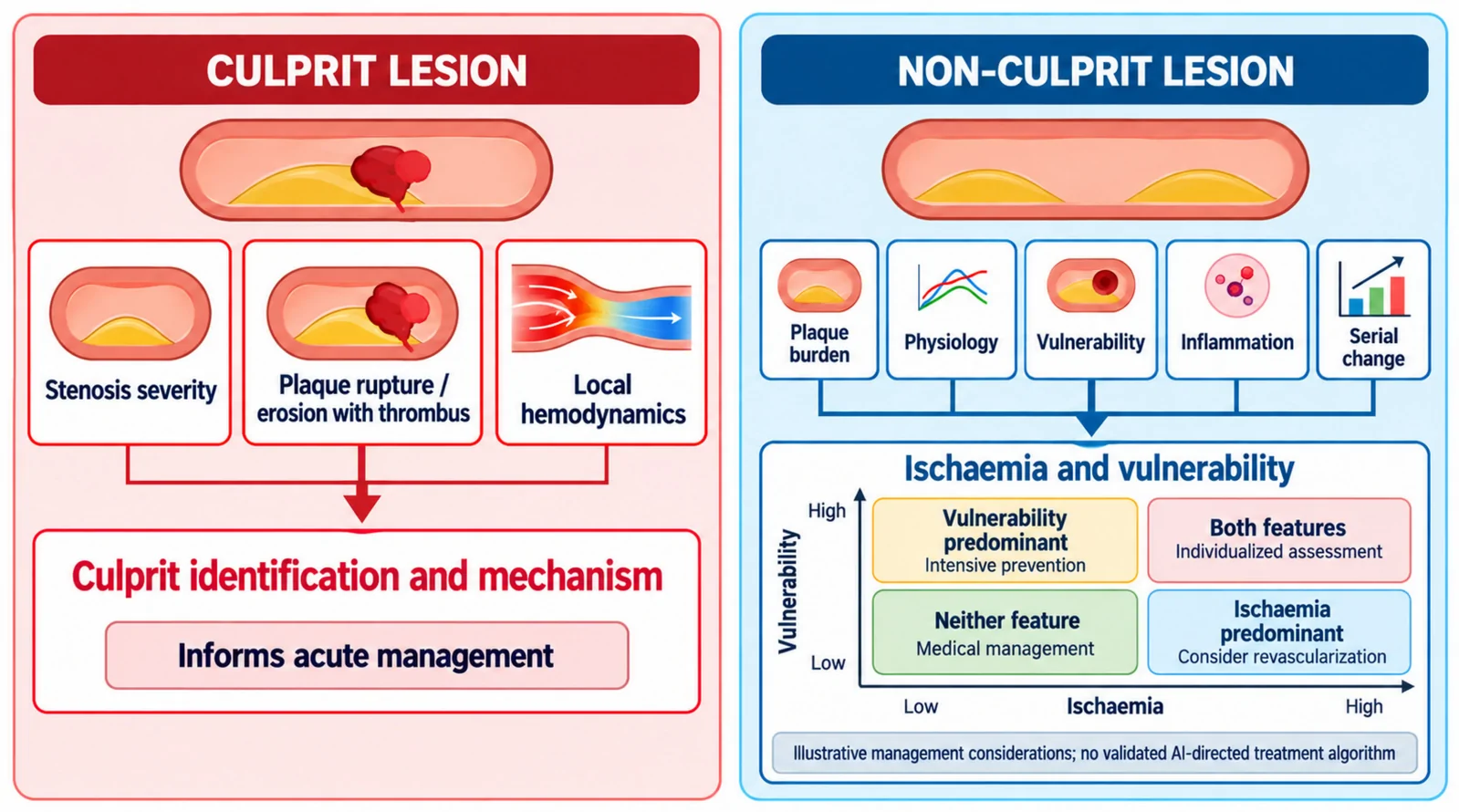
AI-supported assessment of culprit and non-culprit coronary lesions. Culprit-lesion assessment focuses on lesion identification and acute plaque mechanism, whereas non-culprit assessment integrates ischemic significance and biological vulnerability. The illustrated non-culprit phenotypes and management considerations are conceptual; no validated AI-directed treatment algorithm is currently available. Abbreviation: AI, artificial intelligence.

This framework could eventually yield a multidimensional lesion map after ACS: one lesion may be ischemic but morphologically stable and suitable for staged PCI; another may be non-ischemic but vulnerable and warrant intensive systemic prevention; a third may combine both features and represent a higher-priority residual lesion. The latter hierarchy remains hypothesis-generating rather than guideline-based. Natural-history data support risk from non-flow-limiting vulnerable plaques, and PREVENT provides randomized evidence for preventive PCI in a carefully selected imaging-defined population [7,75,99], but no validated AI strategy currently assigns treatment on the basis of this combined phenotype. The complementary assessment of culprit and non-culprit lesions is summarized in Figure 2.

## 8. AI for Procedural Planning and PCI Guidance

### 8.1. From Visual Estimation to Automated Quantitative Coronary Angiography

Invasive coronary angiography remains the anatomical backbone of PCI, but visual stenosis assessment is affected by interobserver variability [100]. Manual quantitative coronary angiography (QCA) is more objective but requires dedicated analysis. AI-QCA aims to provide rapid vessel segmentation, lesion detection, and geometric measurements during catheterization [70,101]. In the validation by Kim et al., the pipeline was trained on 7658 images from 3129 patients and tested on 676 images from 370 patients. It detected 995 of 1115 reference lesions (89.2%); among the 995 matched lesions, 892 (89.6%, equivalent to 80.0% of all reference lesions) had an absolute difference in percentage diameter stenosis of no more than 10% [70]. Correlation was strongest for minimum lumen diameter (R^2^ = 0.864) and reference lumen diameter (R^2^ = 0.840), lower for percentage diameter stenosis (R^2^ = 0.686), and weakest for lesion length (R^2^ = 0.435). Frame selection was manual before automated analysis. False negatives were more frequent in distal segments and low-grade lesions; diffuse disease was not specifically evaluated [70].

A second validation study automated best-frame selection within an investigator-selected angiographic projection and showed strong study-specific agreement for lesion length and reference diameter, with more moderate agreement for percentage diameter stenosis [101]. These systems standardize information present in angiography but cannot recover plaque burden, remodeling, or other biological features absent from lumenography [8,78,79,102].

### 8.2. Potential Relevance to Stent Sizing and Lesion Preparation

Automated reference-vessel diameter and lesion-length measurements can support stent sizing and lesion coverage, and rapid AI-QCA may be valuable where intracoronary imaging is unavailable [70,101]. Device-to-vessel diameter mismatch is associated with lesion-oriented events, but angiography remains an imperfect substitute for IVUS or OCT because plaque burden, positive remodeling, and external elastic-membrane dimensions are not represented by lumenography [8,78,79,102,103]. Intracoronary imaging therefore remains particularly important in calcified, diffuse, left-main, ostial, and bifurcation disease [8,78,79,80].

Prospective procedural evidence is now available. In the multicenter FLASH trial, 400 patients were randomized at 13 sites and 395 were included in the primary analysis. AI-QCA-assisted PCI was non-inferior to OCT-guided PCI for post-PCI minimal stent area (6.3 ± 2.2 versus 6.2 ± 2.2 mm^2^; *p* for non-inferiority < 0.001), although OCT-defined stent malapposition was more frequent with AI-QCA (13.6% versus 5.6%; *p* = 0.007) [72]. Thus, AI-QCA-assisted sizing and guidance has randomized procedural evidence, but no end-to-end multimodal recommendation system has yet demonstrated superiority for clinical outcomes. Automated recommendations for calcium modification, bifurcation strategy, or thrombotic morphology remain investigational.

### 8.3. Human Acceptance Is a Separate Endpoint from Algorithmic Accuracy

Technical agreement does not guarantee acceptance. In a pilot human-factor assessment involving four interventional cardiologists and 60 lesion-level assessments, 43/60 assessments (71.7%) correctly distinguished AI-generated from manual QCA outputs, and 42/60 (70.0%) agreed more closely with manual QCA [101]. These results should be described at the assessment level rather than as the proportion of participating cardiologists. For procedural use, systems should expose contours and measurements, allow rapid operator verification, and communicate uncertainty. Human oversight remains essential when an erroneous measurement could directly affect device selection or treatment [12,13].

## 9. AI for Post-ACS Risk Stratification

### 9.1. Why Post-ACS Risk Is Intrinsically Multidimensional

After successful culprit-lesion treatment, patients remain at risk of recurrent MI, cardiovascular death, progression of residual coronary disease, heart failure, and bleeding related to antithrombotic therapy [3,66,104,105]. Because these events arise through partly different mechanisms, post-ACS management requires separate estimation of ischemic, hemorrhagic, and mortality risks rather than a single generic ‘high-risk’ category [104,105]. Representative studies evaluating ML-based prognostic stratification after ACS are summarized in Table 3.

ML can model more variables and nonlinear interactions than traditional scores, but greater complexity does not guarantee better discrimination. In an early STEMI study, ML models predicted 30-day mortality with AUCs up to approximately 0.91, yet performed similarly to GRACE, and gains plateaued after approximately 15 variables [35]. The principal opportunity for AI may therefore lie in integrating dynamic, time-dependent, or unstructured information that conventional scores cannot capture efficiently, rather than applying a more complex algorithm to the same limited baseline variables [10,11].

### 9.2. Simultaneous Prediction of Death, Recurrent Ischemia, and Bleeding

One of the most clinically relevant ACS applications has been the development of separate ML models for one-year mortality, recurrent MI, and major bleeding. In a pooled derivation cohort of 19,826 patients with an external validation cohort of 3444, the models achieved external AUCs of approximately 0.92 for mortality, 0.81 for recurrent MI, and 0.86 for major bleeding. These levels of performance were maintained when the external cohort was examined over a two-year horizon, indicating reasonable temporal robustness [36].

The most interesting clinical contribution is not the mortality model in isolation but the ability to cross-classify ischemic and bleeding risks. After ACS, intensified antithrombotic therapy may reduce recurrent ischemia while increasing bleeding [105]; therefore, identifying a patient as globally “high risk” is insufficient. A multidimensional model can distinguish patients with high ischemic but relatively low bleeding risk from those in whom both risks are elevated or in whom hemorrhagic risk predominates. Such separation is conceptually closer to the therapeutic decisions clinicians actually make regarding antithrombotic intensity and duration [36].

The models also demonstrated marked gradients in observed events across predicted-risk strata, with the highest-risk decile accounting for substantially greater absolute rates of death, recurrent infarction, and bleeding. This supports their ability to identify clinically distinct risk groups, but does not establish that treatment modification based on these categories improves outcomes. The distinction is essential: risk prediction can identify whom clinicians should worry about, whereas treatment-effect prediction is required to determine which intervention will benefit that individual patient [10,36].

### 9.3. Time-to-Event Modelling

Mortality after ACS is a time-to-event process, whereas binary one-year prediction ignores both event timing and censoring. Survival ML preserves this temporal dimension while accommodating nonlinear interactions that may violate conventional proportional-hazards assumptions [37].

A random survival forest developed in ACSIS and externally tested in MINAP outperformed conventional Cox models and DeepSurv, with a C-index of approximately 0.92 internally and 0.81 externally despite substantial cohort differences [37]. The decrease after transfer underscores the importance of external validation and of matching model complexity to the available data. Continuously updated hazard estimates could inform discharge, follow-up, rehabilitation, and monitoring, but their clinical value requires prospective evaluation.

### 9.4. Dynamic In-Hospital Risk

Scores based on presentation data cannot reflect deterioration or recovery during hospitalization. EHR-based ML can instead incorporate features derived from repeated physiological and laboratory measurements [38].

In 2414 hospitalized ACS patients, an XGBoost model incorporating clinical characteristics and serial measurements achieved an AUC of approximately 0.913 for in-hospital mortality, compared with 0.904 for logistic regression using the same selected variables [38]. The larger improvement arose from the richer longitudinal input rather than algorithmic complexity, suggesting that dynamic data may be more important than model class. However, the study was retrospective, single-center, and lacked external validation; such models may encode institution-specific testing and care patterns and therefore require rigorous transportability assessment.

### 9.5. ECG as a Prognostic Biomarker

The same ECG used for early ACS diagnosis may contain information about long-term prognosis. Conventional clinical scores typically reduce ECG information to a few predefined features such as ST deviation, whereas ML can quantify dozens or hundreds of temporal and morphological characteristics. This creates the possibility that the ECG acts as a high-dimensional digital biomarker reflecting not only acute ischemia but also underlying myocardial substrate, conduction disease, autonomic state, and cumulative cardiovascular injury [10,106].

A prospective chest-pain cohort using 73 morphological ECG features and survival ML provided clinically meaningful separation of longer-term mortality risk in the derivation cohort, and the derived risk groups outperformed the HEART score. In the independent external cohort, validation focused on 30-day mortality; moderate- and high-risk patients had approximately four- and six-fold higher odds of death than the low-risk group [106]. The cohort included patients with suspected rather than exclusively confirmed ACS, which broadens its emergency-department applicability but limits direct extrapolation to a purely post-ACS population.

The broader implication is that a diagnostic test need not have only one clinical function. A single ECG may simultaneously contribute to acute diagnosis, OMI detection, and future risk estimation. This raises the prospect of extracting multiple algorithmic outputs from the same data acquisition without increasing procedural burden. Clinically, however, these outputs must remain task-specific: an ECG model optimized for acute coronary occlusion cannot be assumed to provide calibrated mortality prediction unless it has been separately trained and validated for that endpoint [10,12].

### 9.6. From Risk Prediction to Individualized Secondary Prevention

The clinically meaningful endpoint of post-ACS AI is not a more elaborate risk score but a more effective allocation of preventive therapy. A future system could potentially integrate residual plaque burden, renal function, bleeding history, treatment adherence, LDL-C response, ventricular function, ECG-derived risk, and prior events to continuously estimate recurrent ischemic and hemorrhagic hazards. In principle, such information could support decisions regarding antithrombotic intensity, lipid-lowering escalation, follow-up frequency, rehabilitation, or additional imaging.

However, prognostic models estimate what is likely to happen under observed patterns of care; they do not necessarily predict how risk will change under alternative treatments. This distinction between prognostic prediction and treatment-effect prediction is fundamental. A patient at high risk of recurrent MI may not derive greater benefit from a particular intervention if that same treatment produces disproportionate bleeding or competing harm. Therefore, the next generation of AI should aim to model individualized net treatment benefit rather than merely identifying the highest-risk patients [10,12].

Clinical trials must therefore evaluate not only discrimination and calibration, but also whether AI-informed management improves net cardiovascular outcomes without introducing new forms of overtreatment or inequity [12].

## 10. From Single-Modality Algorithms to Multimodal AI

### 10.1. Why ACS Is Fundamentally a Multimodal Disease State

ACS diagnosis and management are intrinsically multimodal. Symptoms describe the clinical manifestation; ECG reflects electrical ischemia; hs-cTn quantifies cardiomyocyte injury; angiography identifies luminal anatomy; CCTA characterizes plaque and hemodynamics; OCT and IVUS interrogate lesion morphology; coronary physiology estimates ischemic significance; and longitudinal EHR data capture comorbidities, treatment, and subsequent trajectory. No single modality represents the entire disease process, because each measures a different biological layer.

**Figure 3:**
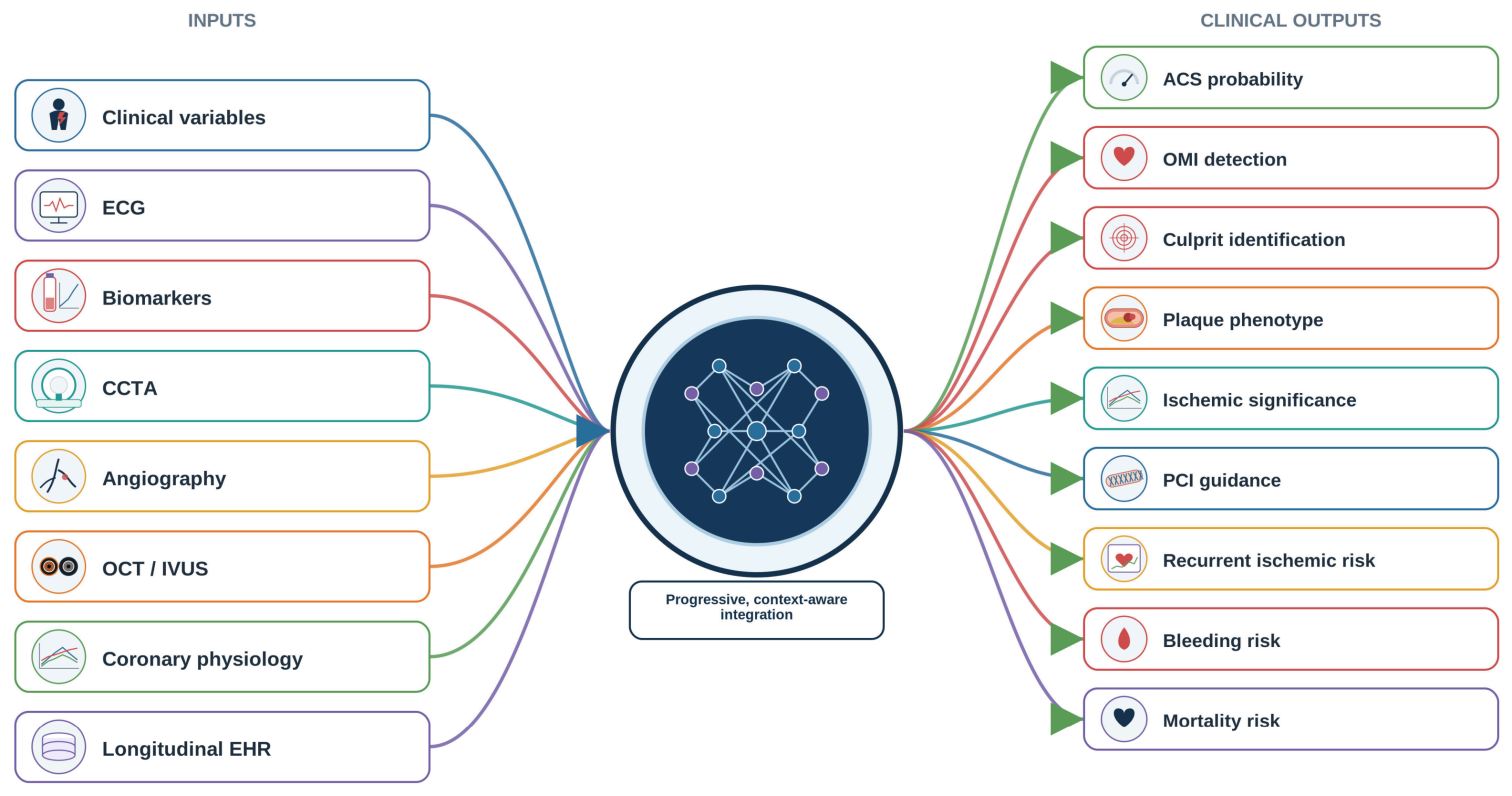
Multimodal AI architecture in acute coronary syndromes. Clinical, electrocardiographic, biochemical, imaging, physiological, and longitudinal data are integrated into task-specific diagnostic, interventional, and prognostic outputs. Abbreviations: ACS, acute coronary syndrome; AI, artificial intelligence; CCTA, coronary computed tomography angiography; ECG, electrocardiogram; EHR, electronic health record; IVUS, intravascular ultrasound; OCT, optical coherence tomography; OMI, occlusion myocardial infarction; PCI, percutaneous coronary intervention.

This explains why some of the strongest single-modality algorithms reach different performance ceilings. DL ECG can identify overt STEMI or OMI with high accuracy, but NSTEMI discrimination improves substantially when troponin is added. Troponin-based algorithms provide excellent probabilistic diagnosis but cannot localize plaque morphology or determine whether a residual stenosis is ischemic. CCTA can identify high-risk lesion phenotypes but does not directly capture the acute electrical or biochemical consequences of plaque disruption. Multimodal AI is therefore not simply an attempt to increase AUC by adding variables; it is an effort to reconstruct ACS from complementary biological representations [5,18,20,26]. The multimodal architecture linking complementary data sources to task-specific diagnostic, interventional, and prognostic outputs is illustrated in Figure 3.

### 10.2. The Difference Between Concatenating Variables and True Multimodal Learning

Many current “multimodal” studies are, in practice, models in which variables from different sources are concatenated into a single structured dataset. This strategy can be clinically useful, as shown by models integrating ECG information, troponin, symptoms, demographics, or routine laboratory results, but it remains conceptually different from architectures that learn representations directly from raw signals and images [46,49].

True multimodal learning attempts to preserve the information structure of each domain before combining them. A raw ECG may be processed by a transformer, CCTA by a convolutional or vision-transformer network, tabular EHR data by a separate encoder, and clinical text by a language model; their learned representations can then be projected into a common latent space. Such shared representations may capture relationships that are difficult to express as manually defined variables–for example, whether a subtle ischemic ECG pattern is concordant with a particular plaque phenotype or physiological distribution [10].

### 10.3. A Clinically Coherent Multimodal Architecture for ACS

A clinically useful system should integrate information progressively as it becomes available: symptoms, vital signs, and ECG at first contact; hs-cTn and laboratory results in the emergency department; CCTA or angiography after imaging; intracoronary imaging and physiology in the catheterization laboratory; and treatment exposure and longitudinal EHR data after discharge [10,11].

This context-aware design should tolerate missing modalities and update predictions as new data arrive. For example, an ECG-derived OMI probability could be recalibrated after troponin, angiography, or intracoronary imaging, rather than requiring a complete fixed input set [10]. The same principle could generate separate lesion-level estimates of ischemia and future instability by sequentially adding anatomy, physiology, and plaque phenotype [25,26,71].

### 10.4. From Task-Specific Models to Foundation Models

Current cardiovascular AI is largely task-specific, with separate models for AMI detection, plaque characterization, FFR estimation, and prognosis. This fragmentation increases the burden of training, integration, validation, and regulation. Foundation models instead aim to learn broad representations from heterogeneous datasets and adapt them to multiple downstream tasks [10].

An ACS foundation model could theoretically integrate ECG, imaging, text, biomarkers, and procedural data to support presentation, culprit-lesion, interventional, and prognostic tasks. This remains a forward-looking concept rather than a validated clinical platform [10]. Even with a shared representation, each output requires dedicated validation; performance in ECG classification cannot establish safety for revascularization guidance or prognosis [12,13].

### 10.5. Multimodal AI Should Augment Rather than Reproduce Clinical Reasoning

The most compelling rationale for multimodal AI is not that clinicians are incapable of integrating ECG, biomarkers, and imaging. Expert ACS care already does this. The computational advantage lies in the ability to preserve high-dimensional information that is ordinarily compressed before human integration: the ECG becomes “ST elevation present/absent,” CCTA becomes “50–70% stenosis with high-risk plaque,” and OCT becomes a small set of categorical descriptors. AI can instead integrate the underlying signal distributions themselves [10,11].

This distinction suggests that the greatest incremental value may come from combining raw data modalities, not simply combining more clinician-derived variables. A model receiving raw ECG waveforms, CCTA volumes, angiographic videos, and longitudinal laboratory trajectories may discover interactions that are lost after manual feature extraction. However, such systems will require very large, carefully linked datasets, and the technical challenge of aligning these modalities at patient, vessel, lesion, and time levels should not be underestimated [10].

The clinical model should therefore remain collaborative. AI can quantify latent patterns and probabilistic relationships, while the clinician contributes causal interpretation, treatment context, patient preference, procedural feasibility, and judgment regarding consequences of false-positive or false-negative recommendations.

### 10.6. The Transition from Prediction to a Continuously Updated ACS Digital Phenotype

The ultimate multimodal concept is a continuously updated digital phenotype rather than a single score. Symptoms and ECG could define the initial state; hs-cTn, angiography, intracoronary imaging, and physiology could progressively refine diagnosis and lesion characterization; and follow-up biomarkers, treatment, and events could update prognosis [10,11].

The system should retain clinically distinct outputs—such as acute occlusion, plaque rupture or erosion, lesion-specific ischemia, procedural outcomes, recurrent MI, bleeding, and mortality—because their therapeutic implications differ [5,26,29,36,72]. Current evidence remains dominated by single-task retrospective or observational models, but key components already exist, including AI-ECG, individualized troponin models, automated plaque analysis, AI-supported intracoronary imaging, computational physiology, automated QCA, and survival prediction [10,12].

## 11. Current Limitations and Barriers to Clinical Translation

### 11.1. Study Design, Reference Standards, and Reporting

The evidence base for AI in ACS remains heterogeneous. A systematic review of 66 studies reported high pooled accuracy for AI-assisted ACS detection, but judged the overall quality of evidence low, largely because case–control designs predominated [39]. Such designs may separate clear cases from healthy controls more easily than an algorithm can distinguish ACS from other causes of chest pain in consecutive emergency patients. Large datasets do not eliminate biased selection, information leakage, or uncertain outcome labels. Future studies should therefore evaluate representative patients at the point where the model is intended to inform care, using independent validation and clearly reported thresholds and clinically relevant performance measures [11,39].

The reference standard itself may also be uncertain. Culprit identification in multivessel NSTEMI can be difficult; OMI is a dynamic state that may change before angiography; and OCT-defined plaque erosion is an imaging phenotype rather than a histological diagnosis [5,13,29,31]. Model performance should be interpreted in light of these limitations in the labels used for training and validation.

### 11.2. Generalizability and Input Quality

Performance may change across healthcare systems, patient groups, ECG devices, troponin assays, scanners, and treatment pathways. External validation should therefore assess both discrimination and calibration in the intended population, followed by monitoring as equipment and practice evolve [11,12,13,107].

For imaging-based AI, technical feasibility matters as much as accuracy in analyzable cases. Motion or calcium-related artifacts on CCTA, incomplete or inadequate OCT/IVUS pullbacks, and unsuitable angiographic projections may prevent reliable analysis [6,68,69,78,79,95,96,97]. Studies should report the proportion of excluded or non-analyzable examinations and technical failures. Clinical systems should assess input quality and withhold a result when the data are inadequate [12,13].

### 11.3. From Model Accuracy to Better Clinical Decisions

A high AUC does not establish that an AI tool improves ACS care. Diagnostic thresholds must reflect the intended action: missing an OMI, for example, has different consequences from an unnecessary cath-lab activation [5,43]. Likewise, an accurate risk estimate does not show that changing treatment on its basis will improve outcomes.

Prospective studies should test the AI-supported clinical strategy, including when the result becomes available, how clinicians respond, and what happens to patients. Relevant outcomes include time to appropriate reperfusion, safe rule-out, procedural decisions, false alerts, downstream testing, and clinical events [5,12,13,44,47]. Integration into existing systems is essential in a time-sensitive pathway; extra processing steps or excessive alerts could undermine an otherwise accurate model [10,11,12,13]. Explainability may help clinicians inspect an output, but it cannot replace validation or establish that the model has learned a causal mechanism [13,108].

### 11.4. Safe and Equitable Implementation

Deployment also requires attention to data protection, control of software updates, clinical accountability, and unequal performance across patient groups [10,11,12,13,39]. These concerns become especially consequential when AI influences access to invasive assessment or treatment. Evaluation should include subgroup performance, clinician oversight, and monitoring after implementation. Costs and unintended increases in testing or procedures must also be measured; faster analysis alone does not establish cost-effectiveness [12,13].

**Figure 4:**
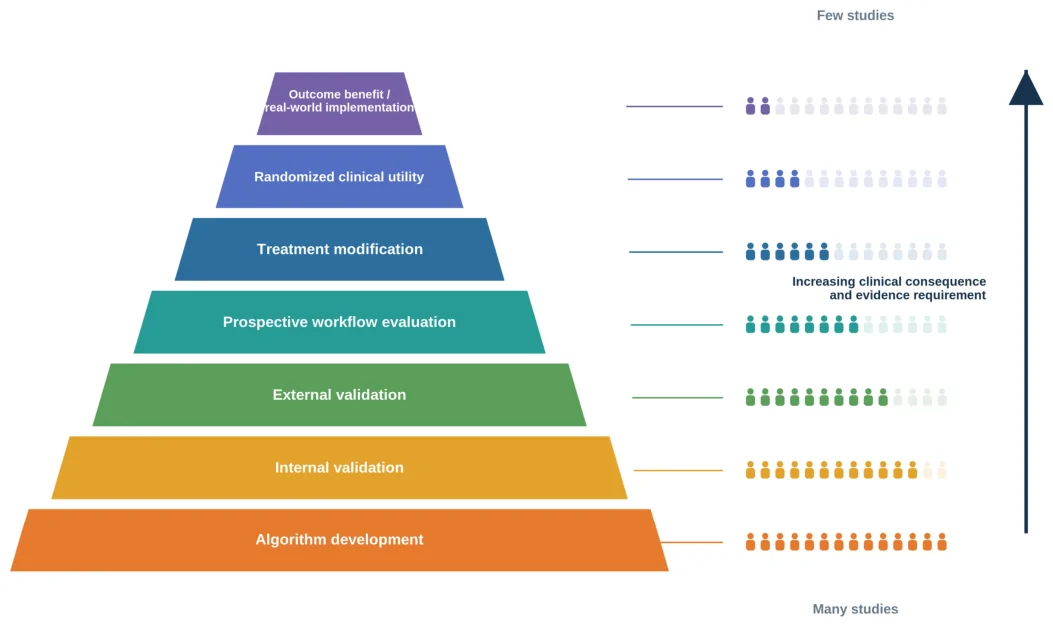
Translational evidence hierarchy for AI in acute coronary syndromes. Evidence progresses from algorithm development and validation to workflow evaluation, treatment modification, randomized clinical utility, and real-world outcome benefit. Abbreviations: AI, artificial intelligence.

The progression from algorithm validation to demonstrated clinical benefit is summarized in Figure 4.

## 12. Conclusions

The greatest potential of AI in ACS lies in connecting information that clinicians must currently interpret across separate stages of care: the presenting ECG and troponin results, coronary anatomy and plaque characteristics, lesion-specific physiology, procedural findings, and subsequent risk. Across these domains, AI has shown promising ability to detect patterns, standardize measurements, and generate patient- or lesion-specific estimates. Applications such as OMI recognition and automated coronary imaging are particularly relevant when they address a time-sensitive or uncertain clinical decision.

The strength of evidence, however, varies considerably between applications. Accurate detection or measurement does not establish that an AI-informed decision will improve treatment or patient outcomes. This distinction is especially important for culprit-lesion identification, non-culprit revascularization, and individualized prevention, where prediction must ultimately be linked to an effective clinical action.

The next priority is prospective evaluation of AI within the workflows in which it is intended to operate. Studies should assess not only accuracy, but also technical failures, effects on clinician decisions, treatment delays, unnecessary interventions, equity, and patient outcomes. AI will be most valuable when it helps clinicians make a better decision at the right time, with uncertainty visible and responsibility for care remaining with the clinical team.

## Figures and Tables

**Table 2:** Selected studies of AI and computational methods for coronary imaging, culprit-lesion characterization, intracoronary imaging, and physiology.

Study	Setting/Sample	Data & Model	Clinical Question	Main Finding	Clinical Relevance/Caveat
Duguay et al., 2017 [23]	ACS + multivessel CAD; 48 patients/81 deferred lesions	CCTA; ML CT-FFR	Non-culprit lesion risk	CT-FFR ≤ 0.80 identified 23/81 lesions and added prognostic information beyond clinical/anatomic risk.	Early hypothesis-generating evidence that residual physiological significance may matter after ACS; small single-center cohort.
Min et al., 2020 [30]	602 lesions/45,400 OCT frames	OCT; DenseNet + Grad-CAM	Automated TCFA detection	Test AUC 0.96; frame accuracy 92.8%; TCFA burden strongly correlated with expert analysis.	Shows rapid automated vulnerable-plaque phenotyping; not ACS-specific.
Cho et al., 2021 [27]	598 patients/598 vessels	IVUS; deep-learning image classification	Attenuated plaque and calcium detection	High diagnostic accuracy with whole-vessel processing in seconds.	Supports real-time automated IVUS phenotyping; mixed CAD population and no outcome-guided strategy.
Milzi et al., 2021 [6]	AMI + multivessel CAD; 220 patients/280 non-culprit vessels	Angiography; QFR	Functional significance of non-culprit lesions	AUC 0.887 vs. staged FFR/CMR/SPECT; outperformed anatomic QCA.	Useful computational physiology evidence, but QFR is not intrinsically an AI method and analysis was retrospective.
Wang et al., 2022 [24]	Serial CCTA; 202 patients/302 lesions	Serial CCTA + CT-FFR; XGBoost	Future ACS from plaque evolution	Dynamic plaque/physiology features yielded AUC 0.918.	Introduces dynamic plaque phenotyping; small retrospective matched cohort.
Park et al., 2022 [29]	ACS culprit-lesion OCT; 581 development + 292 external	Sequential OCT; Transformer	Plaque erosion diagnosis	External lesion-level AUC 0.91; AI assistance increased less-experienced reader sensitivity from 72.1% to 93.0%.	Strong ACS-specific external validation and direct evidence of human-AI complementarity.
Araki et al., 2023 [31]	581 development + 292 external	OCT; Vision Transformer	Layered/healed plaque detection	AUC 0.860 internally and 0.845 externally, outperforming single-frame CNN.	Extends AI from acute culprit morphology to healed instability; expert OCT, not histology, was the reference.
Li et al., 2023 [25]	Prior CCTA in ACS; 132 patients/240 lesions	CCTA + CT-FFR + PCAT; ML	Culprit-lesion prediction	Selected logistic-regression model test AUC 0.819; RF candidate train AUC 0.990 vs. test AUC 0.754.	Integrated multidomain phenotyping with internal temporal validation only; the RF candidate showed overfitting.
Omori et al., 2023 [32]	Prospective validation; 200 patients/253 vessels; predominantly non-AMI	Angiography; ML angioFFR	Prediction of invasive FFR ≤ 0.80	Accuracy 87.7%; sensitivity 76.8%, specificity 94.3%, AUC 0.90.	Only 10.5% had unstable angina; MI within 72 h was excluded.
Koo et al., 2024 (EMERALD-II) [26]	351 patients; 363 culprit/2088 non-culprit lesions	CCTA plaque + hemodynamics; AI quantitative analysis	Future culprit-lesion prediction	Adding AI features improved validation AUC from 0.78 to 0.84.	Key evidence that plaque morphology and hemodynamics are complementary; retrospective lesion-level design.
Narula et al., 2024 (REVEALPLAQUE) [69]	Prospective 15-center validation; 237 patients/432 lesions	CCTA vs. IVUS; AI plaque quantification	Automated plaque measurement	Correlation vs. IVUS: total plaque r = 0.91, calcified r = 0.91, non-calcified r = 0.87.	Important measurement validation underpinning AI-CCTA; stable CAD population, not ACS.
Kim et al., 2024 [70]	3129-patient development; 370-patient validation	Invasive angiography; DL + automated QCA after manual frame selection	Lesion detection and QCA	995/1115 lesions detected (89.2%); 892/995 matched lesions (89.6%; 80.0% of all reference lesions) had |Δ%DS| ≤ 10%.	Technical rather than ACS-specific outcome validation; lesion length had the weakest correlation.
Ullrich-Daub et al., 2025 (QUOMODO) [34]	Randomized ACS multivessel trial; 202 patients/246 non-culprit lesions	Angiography; QFR-guided vs. angiography-guided strategy	Non-culprit revascularization	No significant difference in PCI deferral or 12-month composite clinical outcome.	Important negative randomized evidence: physiological information did not improve this management strategy.
Zimmerli et al., 2025 [33]	High-risk NSTE-ACS; 49 patients/67 non-culprit lesions	CCTA; CT-FFR	Rule-out of significant non-culprit disease	Sensitivity 93%, specificity 79%, NPV 94%, accuracy 85% vs. invasive FFR.	Promising rule-out performance, but a small prespecified post hoc analysis and not strictly AI-specific.
Xu et al., 2025 [71]	Multicenter derivation + prospective validation; total n = 2619	CCTA + CT-FFR; random forest	Culprit identification and future ACS risk	Culprit-lesion AUC 0.85; prospective cohorts showed future-risk discrimination, strongest within ~2 years.	Among the most clinically integrated lesion-level models; prospective cohorts were smaller and one plaque per patient was analyzed.
Kim et al., 2025 (FLASH) [72]	Multicenter RCT; 400 randomized at 13 sites; 395 primary analysis	AI-QCA-assisted vs. OCT-guided PCI	Procedural guidance and stent optimization	Post-PCI MSA 6.3 ± 2.2 vs. 6.2 ± 2.2 mm^2^ (non-inferiority *p* < 0.001); malapposition 13.6% vs. 5.6% (*p* = 0.007).	First randomized procedural evidence for AI-QCA; selected lesions and not powered for long-term clinical superiority.

Abbreviations: ACS, acute coronary syndrome; AI, artificial intelligence; AMI, acute myocardial infarction; AUC, area under the receiver operating characteristic curve; CAD, coronary artery disease; CCTA, coronary computed tomography angiography; CMR, cardiac magnetic resonance; CT-FFR, computed tomography-derived fractional flow reserve; DL, deep learning; FFR, fractional flow reserve; IVUS, intravascular ultrasound; ML, machine learning; NPV, negative predictive value; NSTE-ACS, non-ST-segment elevation acute coronary syndrome; OCT, optical coherence tomography; PCAT, pericoronary adipose tissue; PCI, percutaneous coronary intervention; QCA, quantitative coronary angiography; QFR, quantitative flow ratio; SPECT, single-photon emission computed tomography; TCFA, thin-cap fibroatheroma.

**Table 3:** Selected studies of ML-based prognostic stratification after acute coronary syndromes.

Study	Setting/Sample	Data & Model	Clinical Question	Main Finding	Clinical Relevance/Caveat
Shouval et al., 2017 [35]	ACSIS STEMI registry; *n* = 2782	Clinical variables; RF and other ML models	30-day mortality	Best RF AUC 0.91; performance similar to GRACE and better than TIMI; gains plateaued after ~15 variables.	Useful proof-of-concept, but no external validation and short-term outcome only.
D’Ascenzo et al., 2021 (PRAISE) [36]	19,826 development/internal + 3444 external	Outcome-specific ML models	1-year death, recurrent MI, major bleeding	External AUC 0.92 for death, 0.81 for MI, and 0.86 for major bleeding; enabled joint ischemic/bleeding stratification.	Clinically compelling multidimensional risk model; management based on PRAISE was not prospectively tested.
Hadanny et al., 2022 [37]	ACSIS development; MINAP external validation (206,915)	Random Survival Forest/DeepSurv	1-year mortality	RSF C-index 0.924 internally and 0.811 externally; superior to conventional Cox models.	Large external validation with substantial cross-cohort heterogeneity and missing-data differences.
Li et al., 2023 [38]	Hospitalized ACS; *n* = 2414	Dynamic EHR variables; XGBoost	In-hospital mortality	AUC 0.913; only modest gain over logistic regression using the same selected variables.	Suggests that richer longitudinal data may matter more than algorithmic complexity; single-center, no external validation.
Bouzid et al., 2025 [106]	Prospective chest-pain cohort; 4015 derivation + 3095 external	73 ECG features; Extra Survival Trees	Mortality: longer-term derivation; 30-day external validation	ML-defined risk groups outperformed HEART in derivation and stratified 30-day mortality in external testing.	Demonstrates prognostic information latent in the ECG; population was suspected rather than confirmed ACS.

Abbreviations: ACS, acute coronary syndrome; ACSIS, Acute Coronary Syndrome Israeli Survey; AUC, area under the receiver operating characteristic curve; C-index, concordance index; ECG, electrocardiogram; EHR, electronic health record; GRACE, Global Registry of Acute Coronary Events; MI, myocardial infarction; MINAP, Myocardial Ischaemia National Audit Project; ML, machine learning; PRAISE, Prediction of Adverse Events Following an Acute Coronary Syndrome; RF, random forest; RSF, random survival forest; STEMI, ST-segment elevation myocardial infarction; TIMI, Thrombolysis in Myocardial Infarction.

## Data Availability

No new data were created or analyzed in this study. Data sharing is not applicable to this article.

## Abbreviations

The following abbreviations are used in this manuscript:

ACC	American College of Cardiology
ACS	Acute coronary syndrome
AI	Artificial intelligence
AI-CCTA	Artificial intelligence-enabled coronary computed tomography angiography
AI-ECG	Artificial intelligence-enabled electrocardiography
AI-QCA	Artificial intelligence-based quantitative coronary angiography
AMI	Acute myocardial infarction
AUC/AUROC	Area under the receiver operating characteristic curve
BNP	B-type natriuretic peptide
CAD	Coronary artery disease
CCS	Chronic coronary syndrome
CCTA	Coronary computed tomography angiography
CFD	Computational fluid dynamics
CK-MB	Creatine kinase-myocardial band
CMR	Cardiac magnetic resonance
CNN	Convolutional neural network
CT	Computed tomography
CT-FFR	Computed tomography-derived fractional flow reserve
DICOM	Digital Imaging and Communications in Medicine
DL	Deep learning
ECG	Electrocardiogram
ED	Emergency department
EHR	Electronic health record
EMS	Emergency medical services
ESC	European Society of Cardiology
FFR	Fractional flow reserve
GRACE	Global Registry of Acute Coronary Events
hs-cTn	High-sensitivity cardiac troponin
ICA	Invasive coronary angiography
IHD	Ischemic heart disease
IVUS	Intravascular ultrasound
LDL-C	Low-density lipoprotein cholesterol
MI	Myocardial infarction
ML	Machine learning
NPV	Negative predictive value
NSTE-ACS	Non-ST-elevation acute coronary syndrome
NSTEMI	Non-ST-elevation myocardial infarction
OCT	Optical coherence tomography
OMI	Occlusion myocardial infarction
PACS	Picture archiving and communication system
PCAT	Pericoronary adipose tissue
PCI	Percutaneous coronary intervention
POC	Point of care
PPV	Positive predictive value
QCA	Quantitative coronary angiography
QFR	Quantitative flow ratio
SHAP	Shapley additive explanations
SPECT	Single-photon emission computed tomography
STEMI	ST-segment elevation myocardial infarction
TCFA	Thin-cap fibroatheroma
TIMI	Thrombolysis in Myocardial Infarction
